# The Importance of AGO 1 and 4 in Post-Transcriptional Gene Regulatory Function of tRF5-GluCTC, an Respiratory Syncytial Virus-Induced tRNA-Derived RNA Fragment

**DOI:** 10.3390/ijms21228766

**Published:** 2020-11-20

**Authors:** Eun-Jin Choi, Junping Ren, Ke Zhang, Wenzhe Wu, Yong Sun Lee, Inhan Lee, Xiaoyong Bao

**Affiliations:** 1 Department of Pediatrics, The University of Texas Medical Branch, Galveston, TX 77555, USA; euchoi@utmb.edu (E.-J.C.); juren@utmb.edu (J.R.); wenwu@utmb.edu (W.W.); 2 Department of Chemistry, The University of Houston Clear Lake, Clear Lake, TX 77058, USA; ZhangK6630@uhcl.edu; 3 Department of Cancer Biomedical Science, Graduate School of Cancer Science and Policy, National Cancer Center, Goyang, Gyeonggi-do 10408, Korea; yslee@ncc.re.kr; 4 miRcore, Ann Arbor, MI 48105, USA; inhan@mircore.org; 5 The Institute for Translational Science, The University of Texas Medical Branch, Galveston, TX 77555, USA; 6 The Institute for Human Infections and Immunity, The University of Texas Medical Branch, Galveston, TX 77555, USA

**Keywords:** RSV, tRNA-derived RNA fragments, argonaute, post-transcriptional gene regulation

## Abstract

Respiratory syncytial virus (RSV) is the most common cause of lower respiratory tract infection in infants, the elderly, and immune-compromised patients. It is also a significant contributor to upper respiratory tract infection in the pediatric population. However, its disease mechanisms are still largely unknown. We have recently shown that a tRNA-derived RNA fragment (*tRF*) from the 5′-end of mature tRNA encoding *GluCTC* (tRF5-GluCTC), a recently discovered non-coding RNA, is functionally important for RSV replication and host gene regulation at the post-transcriptional level. However, how tRF5-GluCTC carries out the gene regulation is not fully known. In this study, we found that tRF5-GluCTC has impaired gene *trans*-silencing function in cells deficient of AGO1 or 4, while AGO2 and 3 seem not involved in tRF5-GluCTC-mediated gene regulation. By pulling down individual AGO protein, we discovered that tRF5-GluCTC is detectable only in the AGO4 complex, confirming the essential role of AGO4 in gene regulation and also suggesting that AGO1 contributes to the gene *trans*-silencing activity of tRF5-GluCTC in an atypical way. We also found that the P protein of RSV is associated with both AGO1 and 4 and AGO4 deficiency leads to reduced infectious viral particles. In summary, this study demonstrates the importance of AGO1 and 4 in mediating the gene *trans*-silencing function of tRF5-GluCTC.

## 1. Introduction

Respiratory syncytial virus (RSV), a member of the *Pneumoviridae* family, is a leading cause of respiratory tract illness in infants and children worldwide. Nearly all children have had RSV infection by 2 years of age and reinfection occurs repeatedly throughout life [1,2]. RSV infection also leads to an increase in the morbidity and mortality rate in high-risk adults such as immunocompromised patients and the elderly, resulting in a substantial health burden [3,4,5,6]. Although tremendous effort has been made to develop RSV vaccines and therapeutic agents, there is currently no approved RSV vaccine and treatment [7,8].

The importance of non-coding RNAs (ncRNAs), which do not encode proteins but count for 98% of the human genome, has been demonstrated in many biological processes and diseases [9,10,11,12]. Among them, small ncRNAs (sncRNAs, <200 nt), such as microRNAs (miRNAs), Piwi-interacting RNAs (piRNAs), and recently discovered tRNA-derived RNA fragments (tRFs), have been shown to play a critical role in cancer, cellular stress response, metabolic disturbances and viral infections [13,14,15,16]. Right after the discovery on the induction of tRFs by RSV and their role in RSV replication and associated host responses, the aberrant expression of tRFs was also shown to be associated with the infections of hepatitis B virus (HBV), hepatitis C virus (HCV), and human T-cell leukemia virus type 1 (HTLV-1) [17,18,19]. Overall, the investigation of tRFs on viral infections just began. Our recent studies showed that RSV infection induces several tRFs, which are derived from the 5′-end of a subset of tRNAs, namely tRF5s. Some tRF5s including tRF5-GluCTC have been shown to regulate RSV replication and have gene *trans*-silencing function [17,20]. We have also demonstrated that tRF5-GluCTC uses its 3′-end to target genes, therefore, showing different gene inhibitory mechanism from that of miRNAs. However, the mechanism used by RSV-induced tRFs to control genes and associated biological consequences are still largely unknown.

Argonaute (AGO) proteins, which are highly conserved among species, are central components of RNA-induced silencing complex (RISC) in RNA silencing pathways [21,22,23]. Human AGO proteins are classified into eight family members consisting of four for the AGO clade (AGO1-4), which shows ubiquitous tissue expression, and four for the PIWI clade (PIWIL1-4), which is mainly expressed in the germline [24]. Many sncRNAs have been shown to carry out their mRNA targeting function by binding to AGO members and subsequently guiding AGO members to load mRNA targets for them. miRNAs, the best-characterized sncRNAs, target mRNAs whose sequences are partially complementary to miRNAs in the RISC platform [25,26]. Small interfering RNAs (siRNAs) from the exogenous or endogenous sources are also well known to interact with AGO family members for mRNA cleavage [27,28]. Recently, tRFs were suggested to associate with AGO proteins as well and the types of bound AGO proteins seem to be tRF type- and biological event-dependent [29,30,31,32,33]. However, whether AGO proteins are important for tRFs to carry out the gene silencing functions in viral infection has not been reported.

Herein, we hypothesized that RSV-induced tRFs also depend on AGO proteins for their function in post-transcriptional gene regulation. We tested the hypothesis by investigating the effect of AGO silencing on the gene *trans*-silencing function of tRF5-GluCTC, one of the most abundant RSV-induced tRFs [17], followed by checking the association between the AGO protein(s) and tRF5-GluCTC. Given the importance of tRF5-GluCTC in regulating RSV replication and associated host responses, the newly revealed molecular mechanisms underlying the gene regulatory functions of RSV-induced tRFs have the potential to provide insight into antiviral strategy development.

## 2. Results

### 2.1. Both AGO1 and 4 Contribute to the Gene Trans-Silencing Activity of RSV-Induced tRF5-GluCTC

**Figure 1:**
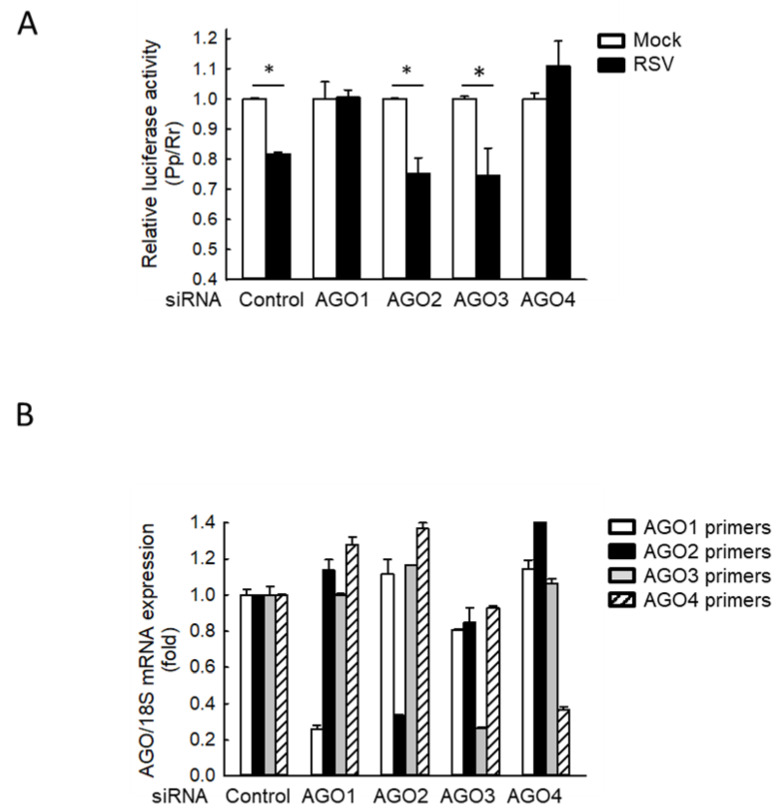
AGO1 and 4 knockdown inhibit RSV-induced tRF5-GluCTC activity. (**A**) A549 cells in triplicate were transfected with 100 nmol/l of siRNA against human AGO1, 2, 3, or 4, or scrambled siRNA (Control) as a negative control in the presence of *Pp*-anti_GluCTC sensor and renilla (*Rr*) plasmids. At 6 h post-transfection, cells were mock- or RSV-infected at a multiplicity of infection (MOI) of 3 and then harvested at 15 h post-infection (p.i.) to measure luciferase activities. *Pp* luciferase values were normalized to *Rr* luciferase values. Data are representative of 3–4 independent experiments and expressed as means ± SE. * *p* < 0.05, relative to the paired white bar. (**B**) Cells were transfected with control or each AGO-specific siRNA as described in (**A**). At 24 h post-transfection, total RNA was prepared, followed by cDNA synthesis and qRT-PCR using each AGO-specific primer. AGO expression was normalized to that of 18S mRNA. Data are representative of three independent experiments and expressed as means ± SE.

Since many sncRNAs, including a few basal cellular tRFs, use AGO proteins to interact with their targets through complete or partial sequence complementarity [33,34], we investigated the effect of AGO depletion on the gene *trans*-silencing function of tRF5-GluCTC. As shown in Figure 1A, in AGO2/3-deficient cells, RSV infection markedly decreased the luciferase activity, which is controlled by a reverse complementary sequence to tRF5-GluCTC in the 3′-untranslated region of the firefly luciferase gene (*Pp*-anti GluCTC), similar to the luciferase suppression by RSV in scrambled siRNA-treated cells (Control), suggesting AGO 2 and 3 are not essential for tRF5-GluCTC to carry out its gene *trans*-silencing function. In contrast, AGO1 or 4 knockdowns impaired RSV-suppressed luciferase, suggesting a role of AGO1 and 4 in mediating the gene *trans*-silencing function of tRF5-GluCTC. Four human AGO proteins are similar in molecular size and exhibit a high sequence similarity in the amino acids (~85%) [22], making it hard for us to discern them by Western blot using commercially available antibodies. Therefore, we used qRT-PCR to confirm the proper suppression of AGO expression by its corresponding siRNA treatment (Figure 1B).

### 2.2. AGO4 Interacts with tRF5-GluCTC

**Figure 2:**
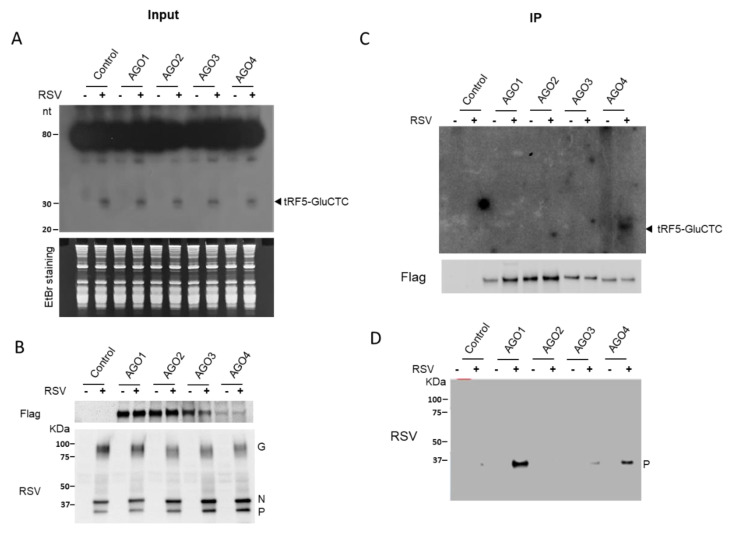
AGO4 interacts with tRF5-GluCTC. (**A**) 293 cells expressing Flag-tagged AGO1, 2, 3, or 4 protein were mock-infected or infected with RSV, at MOI of 1, and total RNA was prepared, followed by Northern hybridization using the tRF5-GluCTC probe to detect the induction of tRF5-GluCTC by RSV infection. EtBr staining is shown for equal loading. EtBr, ethidium bromide. (**B**) Sample input was investigated by Western blot using either an anti-Flag antibody (AGO expression) or an anti-RSV antibody (viral protein expression). (**C**) Total lysates were prepared and followed by IP using an anti-Flag antibody. Flag-AGO bound RNA was purified and subjected to Northern blot for tRF5-GluCTC detection. The AGO expression in the pulldown complex was also investigated by Western blot to ensure the proper IP process. (**D**) To study the association of viral protein(s) with AGO complex, the membrane used in C was stripped and reprobed with an anti-RSV antibody. Data are representative of three independent experiments.

Since the gene *trans*-silencing activity of tRF5-GluCTC was dependent on AGO1 and 4, we then investigated whether tRF5-GluCTC is associated with AGO1 and 4 for gene regulation. The individual AGO protein was immunoprecipitated with an anti-Flag antibody, followed by proteinase K treatment and RNA extraction. Northern hybridization was then used to detect AGO-bound tRF5-GluCTC. Before the immunoprecipitation, we confirmed that tRF5-GluCTC was induced by RSV infection in all AGO-expressing cells (Figure 2A). The proper experimental input and IP were confirmed (Figure 2B). As shown in Figure 2C, tRF5-GluCTC was only detectable in the AGO4 complex, indicating the association of tRF5-GluCTC with AGO4. Surprisingly, we did not observe that tRF5-GluCTC interacted with AGO1, suggesting AGO1 and AGO4 use different mechanisms to contribute to the tRF5-GluCTC-mediated gene regulation.

### 2.3. AGO1 and 4 Interact with RSV-P

It has been recently shown that viral proteins can bind to AGO and regulate the activity and stability of AGO to control viral replication and associated pathogenesis [35,36]. To investigate the possible role of RSV proteins in facilitating the function of tRF5-GluCTC, we investigated whether any viral proteins are associated with the AGO complex. As shown in Figure 2D, RSV-P was strongly detected in the AGO1 and 4 complexes compared with the presence of the P protein in the control, AGO2, or 3 complexes, suggesting that RSV-P may play a role in mediating AGO-dependent tRF5-GluCTC function. To determine whether the interaction of RSV-P with AGOs is virus replication-dependent, we overexpressed the RSV-P protein in the cell lines with constitutively expressed AGO1 or 4. Our IP experiments did not support the interaction of RSV-P with any AGO (data not shown), suggesting that the interaction of RSV-P with AGO1 or 4 is virus replication-dependent.

### 2.4. AGO4 Modulates RSV Replication

**Figure 3:**
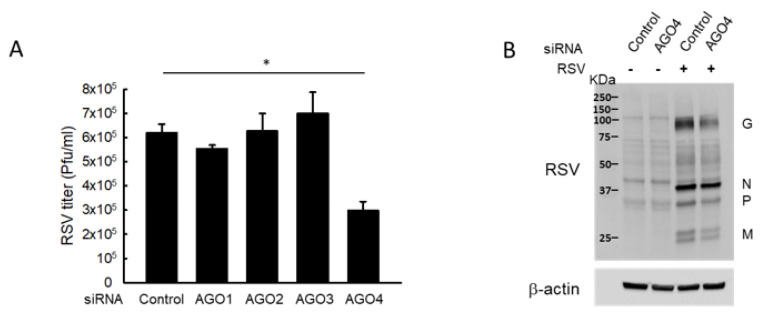
AGO4 knockdown reduces RSV replication. (**A**) A549 cells in triplicate were transfected with 100 nmol/L of siRNA against human AGO1, 2, 3, or 4, or scrambled siRNA (Control). At 24 h post-transfection, the cells were mock-infected or infected with RSV at MOI of 1 for 15 h and harvested for RSV titration assays. Data are representative of three independent experiments and expressed as means ± SE. * *p* < 0.05, relative to the control siRNA. (**B**) Cells, transfected with control or AGO4 siRNA, were infected with RSV and then harvested to prepare total cell lysates, followed by Western blot to check the expression of viral proteins using an anti-RSV antibody. β-actin was used as a control for equal loading of the samples. Data are representative of three independent experiments.

Given the role of tRF5-GluCTC in promoting RSV replication, we investigated whether modulation of AGO1 and AGO4 would affect RSV replication. As shown in Figure 3A, AGO4 deficiency led to a considerable reduction in the infectious RSV yield, compared to the knockdown of other AGOs or control, showing that the importance of AGO4 in RSV replication. Despite the involvement of AGO1 in the gene silencing function of tRF5-GluCTC, there was a minimal reduction in RSV titer of AGO1-deficient cells. The titration result was also confirmed by Western blot showing that the expression of RSV proteins including RSV-G, N, and P was decreased in response to AGO4 knockdown (Figure 3B). These results suggested that the contribution of AGO proteins to RSV replication is complicated by the presence of balance of pro- and anti-viral targets, which are regulated by tRF5-GluCTC and/or other RSV-impacted ncRNAs. Overall, our results demonstrated the importance of AGO1 and AGO4 in mediating the interaction between tRF5-GluCTC and its targets.

## 3. Discussion

This study aims to reveal the molecular mechanisms used by RSV-induced tRF5-GluCTC for its gene *trans*-silencing function. Herein, we demonstrated that AGO1 and 4 are important in controlling the gene-silencing function of tRF5-GluCTC during RSV infection. However, only AGO4 binds to tRF5-GluCTC, suggesting that AGO1 mediates the gene silencing of tRF5-GluCTC differently from AGO4. Our results also showed the interaction of AGO1 and 4 with RSV-P. RSV encodes eleven proteins which are NS1, NS2, N, P, M, SH, G, F, M2-1, M2-2, and L. Among them, RSV-P is a key component of the viral RNA-dependent RNA polymerase (RDRP) complex which is essential to initiate RSV genome replication and mRNA transcription [37]. Our results suggested an additional function of RSV-P being a component regulating the gene silencing functions of RSV-induced tRFs. Overall, this study provides the first evidence on the association of a tRF with AGO protein in viral infection.

tRFs have been recently identified in various cell lines and tissues and implicated in diverse cellular processes such as translation, gene regulation, and apoptosis [38,39,40,41]. Some tRFs have been shown to have a gene *trans*-silencing function using AGO proteins as an interacting partner. The first identification of AGO-bound tRFs was demonstrated in Hela cells by RNA-Seq. The major AGO-associated tRFs are derived from the 5′-end of tRNA, therefore, tRF5s [42]. Subsequent IP analysis showed that tRF5-GlnCTG is associated with AGO1 and 2. In HEK293 cells, the majority of AGO-bound tRFs are derived from the 3′-end of tRNAs (tRF3), which have a preference in binding to AGO3 and 4, but not to AGO1 and 2 [40]. Another report showed that tRF5s and tRF3s are associated with AGO1, 3, and 4, but not AGO2, in HEK293 cells based on photoactivatable-Ribonucleoside-Enhanced Crosslinking and Immunoprecipitation (PAR-CLIP) data [30]. Analysis for short RNA profiles from AGO1-3 IP fractions from THP-1 monocytic cells also identified several tRF5s with dominant interaction with AGO1 [43]. In mature B cells, tRF3-GlyGCC is physically associated with all four human AGOs, with the highest affinity with AGO4 [44]. Overall, unlike miRNAs that commonly use AGO2 as a major AGO protein to attack the targets, tRFs seem not so dependent on AGO2, but other AGO isoforms. Whether virus-induced tRFs interact with AGO proteins has not been shown previously. Herein, we demonstrated that RSV-induced tRF5-GluCTC interacts with AGO4.

**Figure 4:**
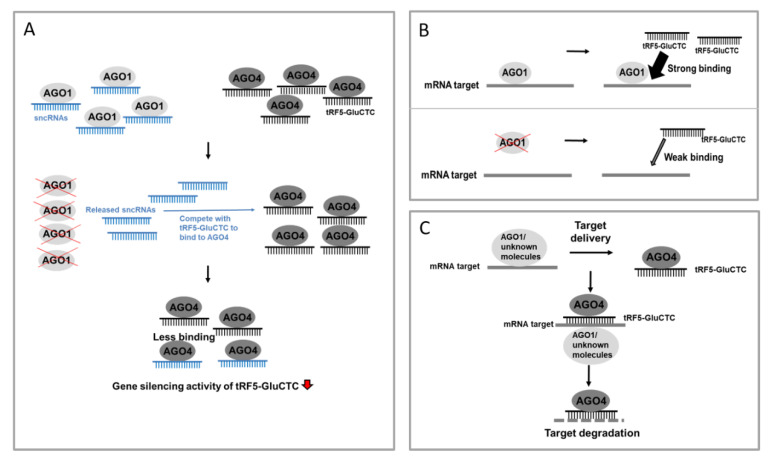
Targeting mechanism models of tRF5-GluCTC. (**A**) In the normal state, RSV-induced tRF5-GluCTC binds to AGO4, leading to an increase in the gene silencing activity. Upon AGO1 depletion, AGO1-associated sncRNAs are released and compete with RSV-induced tRF5-GluCTC for the loading onto AGO4, leading to less tRF5-GluCTC in AGO4 and consequently the reduced gene *trans*-silencing activity of tRF5-GluCTC. (**B**) Upon RSV infection, AGO1 binds to mRNA targets in a tRF5-GluCTC-independent manner, subsequently helping tRF5-GluCTC to find its targets. (**C**) Upon RSV infection, AGO1 or other unidentified molecular partners associate with mRNA targets and deliver them to the AGO4-tRF5-GluCTC complex.

In this study, it seemed that AGO1 regulated the tRF5-GluCTC-mediated gene *trans*-silencing function differently from AGO4, as AGO1 did not bind to tRF5-GluCTC, while AGO4 did. Numerous studies have identified diverse non-miRNA sncRNAs including small nucleolar RNAs (snoRNAs), vault RNAs (vRNAs), and tRFs, to bind to AGO proteins, and different classes of sncRNAs may share common AGO proteins to carry out their gene silencing function [43,45]. Therefore, it is likely for sncRNAs to compete for the loading onto AGO proteins. It has been previously reported that the modulation of tRF expression affects miRNA and siRNA silencing activity [40]. Mutual competition of tRFs and miRNAs on AGO1 loading was also observed [32,46]. Therefore, it is possible that, when AGO1 was knocked down, its associated sncRNAs started loading to AGO4 and competed tRF5-GluCTC off from AGO4, resulting in an indirect effect on gene *trans*-silencing (model shown in Figure 4A).

Recently, it has been shown that AGO also can have its binding preference within target mRNAs, independent of miRNA’s guidance and miRNA-AGO interaction [47]. The observation is also supported by a recent study, which showed that, in *D. melanogaster*, AGO1 can interact with the mRNA targets of miRNAs through the Smaug RNA-binding protein. The target loading to AGO1 does not need miRNA and AGO1-miRNA interaction [48]. Therefore, it may not be necessary for tRF5-GluCTC to bind to AGO1 to guide the AGO-target association. However, AGO1 deficiency-caused target loading impairment and disabled the gene-silencing function of tRF5-GluCTC. The targets in AGO1 are possibly recognized by free cellular tRF5-GluCTC (model demonstrated in Figure 4B) or tRF-GluCTC bound to molecular partners including AGO4 (see model pictured in Figure 4C). In the future, we will investigate which mechanism(s) contributes to AGO1-mediated gene silencing of tRF5-GluCTC.

Overall, our study demonstrated that AGO1 and AGO4 have distinct ways of mediating tRF5-GluCTC-controlled gene regulation. As shown in Figure 3, only AGO4 silencing leads to reduced RSV replication, suggesting that tRF5-GluCTC and other RSV-impacted ncRNAs have more anti-viral targets than pro-viral targets in AGO4. As mentioned, the regulatory functions of tRFs are emerging in viral infection. However, our understanding of tRF functions and regulatory mechanisms in virus infections remains limited. To our best knowledge, the functions of tRFs are only reported for RSV, human immunodeficiency virus type 1 (HIV-1, HTLV-1, HBV, and HCV infections [17,18,19,49].

In the present study, we discussed the platform used by RSV-induced tRF5-GluCTC for its gene targeting function. Given many targets of tRF5-GluCTC are involved in the regulation of RSV replication and host responses [50,51,52,53], these results could provide potential as a novel approach to develop anti-viral interventions against RSV disease.

## 4. Materials and Methods

### 4.1. Cell Lines, Virus, and Antibodies

A549 (human alveolar type II-like epithelial), 293 (human embryonic kidney epithelial), and HEp-2 (human epithelial type 2) cells were purchased from the ATCC (Manassas, VA, USA) and maintained as previously described [50,54]. For AGO-expressing cell lines, 293 cells were transfected with pIRESneo-FLAG/HA-AGO1, 2, 3, 4, or control empty vector and incubated in the media containing 100 mg/mL G418. After 14 days, single-cell cloning was picked up for expansion and prepared for the experiments after the expression confirmation. RSV long strain was propagated in HEp-2 cells at 37 °C and purified by sucrose gradient as previously described [54,55]. Viral titer was determined by immunostaining in HEp-2 cells using polyclonal biotin-conjugated goat anti-RSV antibody (Bio-Rad, Hercules, CA, USA) and streptavidin peroxidase polymer (Sigma-Aldrich, St. Louis, MO, USA), as previously described [54,55]. Primary antibodies against β-actin and Flag were from Sigma-Aldrich and goat anti-mouse IgG-HRP was purchased from Santa Cruz Biotechnology (Santa Cruz, CA, USA).

### 4.2. Quantitative Real-Time PCR (qRT-PCR)

A549 cells were transfected with scrambled siRNA (control) or siRNA against AGO1, 2, 3, or 4, and total cellular RNA was prepared using TRIzol reagents (Thermo Fisher Scientific, Waltham, MA, USA), followed by reverse transcription into cDNA using iScript™ cDNA Synthesis Kit (Bio-rad) according to the manufacturer’s instruction. qRT-PCR was performed using iTaq™ Universal SYBR Green Supermix (Bio-rad) in the CFX Connect Real-Time PCR System (Bio-rad) and the expression of each AGO was calculated using the 2^−ΔΔct^ method. The information on RT primers used to quantify AGOs and 18S is shown in the Supplementary Table S1.

### 4.3. Reporter Gene Assays

The effects of AGO down-regulation on tRF5-GluCTC-mediated gene silencing were investigated using a luciferase reporter system which was developed in our previous study [17]. The firefly plasmids contain a reverse complementary sequence to tRF5-GluCTC in the 3′-untranslated region of the firefly luciferase gene (*Pp*) (*Pp*-anti_GluCTC) and we used this to evaluate the expression or function of tRF5-GluCTC. Previously, we have shown that RSV-induced tRF5-GluCTC suppresses the luciferase expression [17]. If a particular AGO protein is essential for tRF5-GluCTC to carry out the gene silencing, the AGO deficiency should ease the suppressed luciferase expression. In these experiments, we always included a plasmid expressing renilla (*Rr*) luciferase for normalization.

In brief, A549 cells were co-transfected with *Pp*-anti_GluCTC sensor plasmids (firefly plasmids), pRL-CMV plasmids expressing renilla (*Rr*) luciferase (for normalization purpose), and scrambled (control) siRNA or siRNA specifically against AGO using Lipofectamine 2000 (Life Technologies, Grand Island, NY, USA), as previously described [17]. At 6 h post-transfection, cells were mock-infected or infected with RSV at a multiplicity of infection (MOI) of 3 and, at 15 h post-infection (p.i.), lysed to measure luciferase reporter activity using a Dual-luciferase kit (Promega, Madison, WI, USA).

### 4.4. Immunoprecipitation (IP) Assays

AGO-expressing 293 cells, mock-infected or infected with RSV at MOI of 1. At 15 h p.i., the total cell lysates were prepared after the UV crosslinking (UV Stratalinker 1800, Stratagene/Agilent, Santa Clara, CA, USA) and harvested using cell lysis buffer (IP assay kit, Roche, Indianapolis, IN, USA) in the presence of RNase inhibitors. Samples were pre-cleared using protein A/G agarose beads and incubated with an anti-Flag antibody for 1 h at 4 °C. After the addition of protein A/G agarose beads, samples were incubated overnight at 4 °C. The IP complexes were washed using buffers provided by the kit and then treated with proteinase K (New England BioLabs, Ipswich, MA, USA), followed by RNA extraction using Trizol. Associated RNAs in Flag-tagged AGO complexes were analyzed by NB for tRF5-GluCTC. Small amounts from the IP complexes were aliquoted and followed by Western blot using anti-RSV antibodies. The membrane was stripped and reprobed with anti-Flag antibodies to ensure the proper IP process.

### 4.5. Northern Blot

Northern hybridization for tRF5-GluCTC was performed using a tRF5-GluCTC-specific probe as previously described [17,20]. Briefly, RNA was separated in 15% denaturing polyacrylamide gel with 7M urea and then transferred to a positively charged nylon membrane (Amersham Biosciences, Piscataway, NJ, USA). The membrane was hybridized with ^32^P-labeled probes in ULTRAhyb-Oligo solution (Life Technologies, Grand Island, NY, USA) and washed three times according to the manufacturer’s instruction, followed by image development.

### 4.6. Western Blot

The cell lysates were prepared followed by protein quantification using a kit from Bio-Rad, followed by fractionation using SDS-PAGE denaturing gels, as previously described [54]. The separated proteins were then transferred to polyvinylidene difluoride membranes. Membranes were blocked with 5% milk in Tris-buffered saline (TBS)-Tween 20 and incubated with the proper primary antibodies according to manufacturer’s instructions.

### 4.7. Statistical Analysis

Statistical significance was determined using analysis of variance (ANOVA). A *P* value of less than 0.05 was considered significant. Data are presented as means ± standard error (SE).

## Supplementary Materials

The following are available online at https://www.mdpi.com/1422-0067/21/22/8766/s1. Supplementary Table S1. The primer information for qRT-PCR of AGOs and the internal control 18S.
