# Supplementary material for: The Importance of AGO 1 and 4 in Post-Transcriptional Gene Regulatory Function of tRF5-GluCTC, an Respiratory Syncytial Virus-Induced tRNA-Derived RNA Fragment
Source: Int J Mol Sci. 2020 Nov 20;21(22):8766. doi: 10.3390/ijms21228766 (PMC7699471; doi:10.3390/ijms21228766)
Supplement: Supplementary file 1 [file ijms-21-08766-s001.pdf]

Supplementary Table S1. Primers for qRT-PCR

Primer Name	Sequence (5' to 3')
AGO1 forward	CAGTGGACACCAACATCACC
AGO1 reverse	AAACGGTTGTCATCCCAAAG
AGO2 forward	CCGGCCTTCTCTCTGGAAAA
AGO2 reverse	GCCTTGTAAAACGCTGTTGCT
AGO3 forward	ATCCCAGCTGGAACAACAGT
AGO3 reverse	GCGTACGTAAGTGTGGCAGA
AGO4 forward	AGTTGCTTGTTTTGCACCTCAGA
AGO4 reverse	ATTTTACGCAGCTGGTCAGTGA
18S forward	ACATCCAAGGAAGGCAGCAG
18S reverse	TCGTCACTACCTCCCCGG
